# Patterns of childhood tuberculosis diagnosis in Ethiopia: A multicenter cross-sectional study

**DOI:** 10.21203/rs.3.rs-3758745/v1

**Published:** 2023-12-29

**Authors:** Kedir Usmael, Tsegahun Manyazewal, Hussen Mohammed, Getnet Yimer, Lemessa Oljira, Kedir Teji Roba, Tesfahunegn Hailemariam, Tigist Adjeme, Dagmawit Tesfaye, Haileleul Bisrat, Esther Ngadaya, Yimtubezinash Woldeamanuel

**Affiliations:** Dire Dawa University; Addis Ababa University; Addis Ababa University; Addis Ababa University; Haramaya University; Haramaya University; Addis Ababa University; Addis Ababa University; Addis Ababa University; Addis Ababa University; National Institute for Medical Research; Addis Ababa University

**Keywords:** Tuberculosis, children, diagnosis, chest X-ray, Xpert MTB/RIF, Ethiopia

## Abstract

**Background:**

Children share 12% of the global 10 million people infected with tuberculosis (TB) each year. Closing case detection gap in children remains difficult, with 56% of all children and 65% under-five with TB missed each year. We aimed to assess the patterns of childhood TB diagnosis and underlying determinants in Ethiopia when different TB diagnostic platforms are applied.

**Methods:**

A multi-site, cross-sectional study was carried out in Ethiopia as part of the larger EXIT-TB study - evidence-based multiple focused integrated intensified TB screening package. Outpatient children aged ≤ 15 with cough of any duration seeking care at four healthcare facilities in Ethiopia were enrolled consecutively. Participants underwent sputum Xpert MTB/RIF and/or smear microscopy and posteroanterior chest X-ray (CXR), and their clinical and sociodemographic data were captured using a structured questionnaire. Data were analyzed using Stata version 23. Multiple regression model was computed to determine the factors that influence TB case detection, with a 95% confidence interval (CI) and *p < 0.05* taken as statistically significant.

**Results:**

A total of 438 children were enrolled. Of these, 399 had CXR examination of which 55 (13.8%) were suggestive of TB, 270 had Xpert MTB/RIF testing of which 32 (11.9%) were positive, and AFB smear microscopy was done for 51 children of which 2 (3.9%) were positive. Febrile children were more likely to be diagnosed with pulmonary TB than those without fever [aPR = 1.3, 95% CI (1.1–1.4)], and those with a TB contact history were more likely to be diagnosed with pulmonary TB than those with no such contacts [aPR = 1.2, 95% CI (1.1–1.3)]. Children from rural residences were more likely to be diagnosed with TB than those from urban residences [aPR = 1.3, 95% CI (1.1–1.5)].

**Conclusion:**

The findings showed that **c**linical diagnosis remains an important method of TB diagnosis in children and the preferred choice to avert underdiagnosis. A more sensitive TB diagnostic method for children was symptom screening, followed by CXR and Xpert MTB/RIF assay or smear microscopy. Hence, an algorithm that combines clinical, CXR, and microbiological confirmatory tests can improve the rate of pulmonary TB diagnosis in children till more accurate and cost-effective diagnostic tools are accessible. Fever, weight loss, and TB contact history are highly associated with TB positivity rates in children.

## Introduction

Globally, tuberculosis (TB) remains a major cause of death among infectious diseases, with an estimated 10.6 million people falling ill and 1.3 million dying of TB in 2022 [1]. In the last four decades, the emergence of drug-resistant TB (DR-TB) strains and complications associated with human immunodeficiency virus (HIV) co-infection increased the incidence of TB and made TB diagnosis and treatment more problematic [2, 3]. Children have been considered a priority in the 2035 global End TB strategy [4]. Globally in 2022, 16% of the HIV-negative and 18% of HIV-positive people who died from TB were children aged < 15 years [1]. Available data on children suggests limited access to diagnosis and treatment and a higher risk of developing TB among exposed infants and young children [1, 5, 6]. Worldwide, There is a large gap in case detection among children. This age group is most at risk of severe forms of TB and delays in diagnosis can lead to death [7].

According to the WHO 2023 progress update, Ethiopia has almost reached the second End TB Strategy milestone, with rapid reduction of TB mortality rates by 34% [8]. However, the country remains one of 30 high TB burden countries and TB incidence was reported at 119 per 100,000 population in 2021. Despite the availability of rapid microbiological detection methods such as Xpert MTB/RIF, over a half of children are treated for TB based on clinical criteria alone [8]. This underestimates the true nature of TB in children and leaves them vulnerable. In order to correctly diagnose and treat pediatric TB, the performance of the microbiological confirmatory diagnosis modality of pediatric TB needs to be improved through the use of more sensitive and specific diagnostic techniques.

Children less than 7 years of age cannot expectorate sputum specimens properly for laboratory examinations, which calls for more accurate diagnostic techniques and algorithms. TB in children up to 10 years is mostly primary while above 10 years is similar in clinical and radiological presentation to the patterns seen in adults. WHO has adopted standards for TB that emphasize the importance of clinical, radiological and epidemiological data consistent with TB and bacteriological confirmation where possible to diagnose the disease in children. Although chest radiography (CXR) has recently been promoted and recommended by the WHO as a useful tool for TB screening and triage algorithms, access to the instrument and timely diagnosis remain limitations [9–12].

Studies done in Ethiopia reported the prevalence, impact, and molecular epidemiology dynamics of childhood TB [13–20]. However, information is scarce about the potential role and contributions of implementing alternative clinical, radiological, and microbiological TB diagnostic platforms for childhood TB case findings. Hence, this study aimed to determine the pattern of TB diagnosis in children in selected public health facilities in Ethiopia and their determinants as evaluated using different diagnostic platforms.

## Methods

### Study design and setting

The study was a facility-based cross-sectional study using secondary carried out in Ethiopia as part of the larger EXIT-TB study (Evidence-Based Multiple focus Integrated Intensified TB Screening package). The current study focused selectively on TB in children in Ethiopia. EXIT-TB is a larger multi-country intervention study that involved intensified passive TB case finding (screening all patients at the Outpatient departments who passively report any cough irrespective of duration); integrating TB case finding activities into reproductive and child health clinics and diabetics clinics; screening for TB irrespective of symptoms among HIV/AIDS infected individuals with advanced diseases attending Care and Treatment Centers; and targeted contact tracing for all TB patients with child household members. Briefly, the EXIT-TB intervention package involved: I) Screening for TB for all individuals who passively report a cough at OPD and RCH using CXR. Pregnant women with a cough attending RCH were tested using GeneXpert. Those with abnormal CXR were subject to either GeneXpert or sputum smear microscopy depending on the availability of GeneXpert machines in the selected facilities. II) Testing for TB irrespective of symptoms among HIV clinic attendees with advanced diseases and diabetic clinics using GeneXpert. III) Conducting household contact tracing of children with a household member with TB and symptoms screening followed by CXR and those with symptoms and/or abnormal CXR further diagnosed using TB score chart, GeneXpert, or smear microscopy depending on the availability of the GeneXpert machine.

The settings of the current study were four health facilities that were selected from two national regional states (Oromia and Harari) and two city administrations (Addis Ababa and Dire Dawa). These facilities were randomly selected from respective stratified settings by urban and rural. In summary, Chelenko Primary Hospital was from Oromia National Regional State, Hiwot Fina Specialized University Hospital from Harari Regional State, Zewditu Memorial Hospital from Addis Ababa, and Melka Jebdu Health Center from Dire Dawa City Administration. Hiwot Fina Specialized University Hospital and Zewditu Memorial Hospital were from an urban setting whereas Chelenko Primary Hospital and Melka Jebdu Health Center were from rural settings. Hence, in the current study, all four study sites were included and the necessary children-based data was pooled to address the study objectives.

### Participants

Study participants were children (age < = 15) with cough of any duration who visited either of the study facilities. In the collection of the data, healthcare providers linked participants with cough of any duration to the data collectors, requested consent or parental consent based on the participant’s age per the Ethiopian and WHO ethics guidelines. Trained data collectors checked and identified eligible participants, including eligibility for CXR screening procedure and enrollment into the study. Those TB patients who are on anti-TB treatment before the start of the study and those who are diagnosed in other facilities and came to the study facilities for anti-TB treatment services were excluded.

### Data collection and quality control

Data were captured on three key areas: I) Sociodemographic characteristics of study participants: Age, sex, name and type of the healthcare facility attended, residence, and any co-infections including HIV. II) TB clinical symptom screening using the WHO TB symptom screening tool. III) TB diagnostic modality and algorithm, thus the diagnostic modality used to investigate further or confirm TB, including CXR, AFB sputum smear microscopy, Xpert MTB/RIF assay, TB liquid or solid culture, and DR-TB susceptibility testing as applicable.

When patients visit study health facilities, they were screened and recruited immediately as they fulfill the eligibility criteria. The date of health facility visits, first dates of symptom (cough), and dates of diagnosis were captured using the study questionnaire. CXR was offered to the participants stratifying by cough duration of < 2 weeks and ≥ 2 weeks. Sputum specimens were collected and microbiological confirmatory tests were performed among those with CXR results of TB suggestive for < 2 weeks cough duration and those with CXR results of all types (normal, TB suggestive, and non-TB suggestive) for ≥ 2 weeks cough duration.

A strong emphasis was given to keeping the quality of CXR reading and CXRs read by radiologists. For health centers that lack CXR machines, patients were transported to a nearby public healthcare facility with a CXR machine and the study covered the cost. Microbiological confirmation was as per the national TB guideline with either GeneXpert mycobacterium tuberculosis (MTB)/RIF assay or acid-fast bacilli (AFB) microscopy.

### Statistical Analysis

Data were analyzed using Stata version 23. We assessed the screening algorithms with the yields of pulmonary TB cases diagnosed. We calculated the pattern of pulmonary TB cases diagnosis among the presumptive TB cases obtained from children screened for cough of any duration using symptom and/or chest X-ray screening at study health facilities. We assessed the factor that determined the diagnosis of TB cases using multiple regression model with 95% confidence interval (CIs) and p < 0.05 was taken as statistically significant.

### Ethical Considerations

This study was approved by the Institutional Review Board of the College of Health Sciences, Addis Ababa University, and the Institutional Health Research Ethics Review Committee of the College of Health and Medical Sciences, Haramaya University. Written consent was obtained from each participant or parents as applicable, and assent was sought from children under the age of 18. All patients received standard care according to national guidelines and those diagnosed with TB were linked to TB treatment clinics.

### Definition of Terms

#### Presumptive TB cases

patients with cough of ≥ 2 weeks with any chest X-ray results, cough < 2 weeks with chest X-ray abnormality suggestive of TB, and cough of any duration for pregnant women, ART, and diabetic patients with or without the presence of night sweats, fever, hemoptysis or loss of weight were presumptive TB cases who were eligible to be evaluated

#### Pulmonary tuberculosis (PTB)

a participant with lung TB confirmed by Xpert/smear microscopy or clinically diagnosed as per Ethiopian national TB guidelines.

#### Bacteriologically-confirmed TB case

a patient from whom at least one sputum was positive for mycobacterium TB either by Xpert/smear microscopy

#### Clinically diagnosed TB case

participant who did not fulfill the criteria for a bacteriologically confirmed case, but was diagnosed with TB by an experienced clinician and given a full course of TB treatment.

## Results

### Characteristics of study participants

Of 438 study participants, two hundred thirty-two 232/438 (53%) were females and two hundred and six 206/438 (46.8%) were males with a mean age of 7.48 years with a range of 13 years. Age was categorized into three groups; 1–5,5–10 and 10–14 years old (Kefyalew et al,2022). About 155/438 (35.4%) children were less than or equal to 5 years, 178/438 (40.6%) were 5–10 years old and 105/438 (24.0%) were 10–14 years. TB screening was done for 235/438(53.7%) residents of rural and 201/438(45.9%) resident of urban children (Table 1).

### Clinical Features of the Study Participants

Cough of any duration was reported in all 438 of children. Additional clinically assessed TB symptoms include hemoptysis, fever, weight loss, and night sweats. Fever was the most prevalent clinical manifestation and documented in 241/438 (55.1%) of children, followed by TB contact history 231/438 (52.7%) and weight loss 195/438 (44.5%). Bloody sputum was reported only in 16/438 (3.5%). Night sweating and a history of chronic disease were reported in 71/438 (16.2%) and 6/438(1.4%) respectively (Table 2).

### Diagnostic algorytm and pattern of TB diagnosis in children

Among total 438 data of children who sought health care at four health facilities in Ethiopia with cough of any duration; Chest x-ray was done for 399/438 (91%) participants. All view of the chest x ray was Anteroposterior (AP). About 55/399(13.78%) were suggested for TB by chest X-ray of which 36 confirmed bacteriologically. Of 399/438 (91%) presumptive TB cases that had CXR interpreted results, 344/399(86.2%) and 55/399(13.78%) had non-TB suggestive and TB suggestive chest x-ray results, respectively (Table 3).

**Figure 1:**
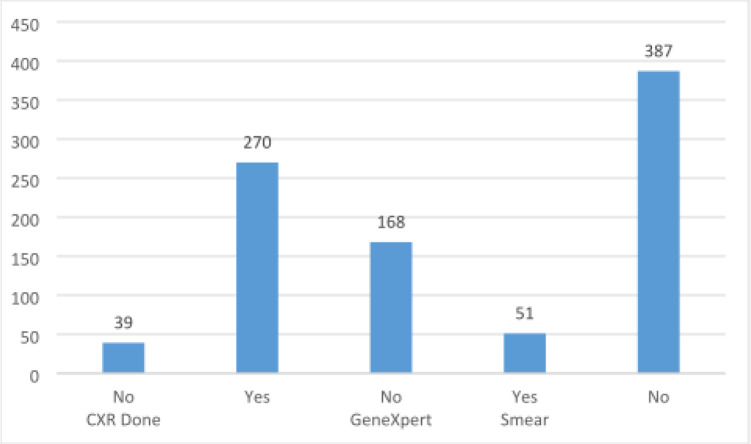
Diagnostic tests done to diagnose TB in children among presumptive TB case in children at health Facilities, Ethiopia

GeneXpert of was done for 270/438(61.6%) of which 32 were positive with a positivity rate of 11.85 whereas GeneXpert from stool sample was done for 75 children of which 4/75(5.3%) were positive. AFB smear was done only for 51 children and only 2 samples was positive with a positivity rate 2/51(4%) (Fig. 1).

The Sensitivity and specificity of chest x-ray, GeneXpert and AFB smear were 17.41% and 70.13%,7.98% and 86.76% and 1.05% and 98.96% respectively.

### Diagnostic Algorithm

**Figure 2:**
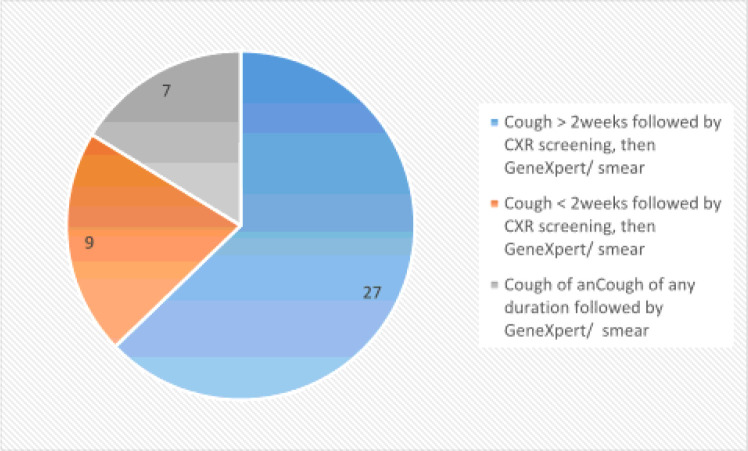
Diagnostic algorithm used to diagnose TB in children among presumptive TB case in children at health Facilities ,Ethiopia

On cough duration and diagnostic tests to confirm TB cases, three different algorithms were used. The first algorithm was cough of more than or equal to 2 weeks followed by chest X-ray screening with any results followed by Xpert /AFB smear microscopy and with this algorithm, we found 63% PTB cases. The second algorithm was the cough of less than 2 weeks followed by chest X-ray screening with TB-suggestive results followed with GeneXpert /smear microscopy and with this algorithm we found 21% PTB cases. The third algorithm was cough of any duration followed by Xpert/smear microscopy and with this algorithm 16% of TB is diagnosed (Fig. 2).

### Factors that determines Pattern of Tuberculosis diagnosis in children

From socio-demographic characteristics, age group from 5 to 10 years old and residence rural was a statistically significant predictor for the diagnoses of TB diagnosis, among children screened and tested for TB. Children age group 5–10 years are (APR = 2.2, 95% CI:1.5–2.3) times as likely to be diagnosed with PTB as age group < 5 years. Children from rural residence are (APR = 1.3,95% CI:1.1–1.5) times more likely diagnosed than that of urban residence.

Among clinical characteristics, children with a sign and symptoms of of fever were (APR = 1.3 95% CI:1.1–1.4)times more likely to be diagnosed with TB than children with no fever. Additionally, Children with history of contact with TB case in house hold were were (APR = 1.2,95% CI 1.1–1.3)times more diagnosed with PTB than those who had no history of of contact with TB Case (Table 4).

## Discussions

This study examined patterns of TB diagnosis among children in Ethiopia’s public health facilities. The diagnostic pattern was evaluated using three groups of variables; Sociodemographic characteristics of study participants, screening for clinical symptoms, diagnostic modality, and diagnostic algorithm.. Among the socio-demographic characteristics examined, tuberculosis is more frequently diagnosed in young children between the ages of 5 and 10 than in children under 5 and over 10 years of age. This can be explained by the fact that children start school at this age, which increases their exposure to different environments, which increases the risk of infection at this age. The disease, which is also more likely to occur in rural than urban areas, can be explained by poor access to medical care, low vaccination rates and socio-cultural problems, as well as living conditions in rural areas that contribute to the spread of tuberculosis [21, 22].

Clinical signs of suspected TB in children included fever, and were more likely to be diagnosed with PTB than children without fever, and children with a contact history were more likely to be diagnosed with PTB have a higher likelihood of being diagnosed with PTB than those with no contact history. While contact history is less useful in countries with high TB endemic rates [23, 24] an important consideration is that children contract TB in the household in which they live. can be easily traced, unlike adults with many. For example, one study shows that it is not difficult to diagnose multidrug-resistant tuberculosis (MDR-TB) in children, since exposure to adults with MDR-TB is essential to establish this presumptive diagnosis [6]. This result supports the findings of another studies that clinical diagnosis is essential in the management of childhood tuberculosis [7, 25], which is why improving the competence of frontline health workers in the priority in most countries as clinical diagnosis of childhood tuberculosis commonly used in clinical practice [26–29].

Based on the duration of the cough, three different algorithms were used to detect cases of tuberculosis in children. The first algorithm is a cough for ≥ 2 weeks, followed by a chest x-ray with all findings, followed by GeneXpert/AFB swab microscopy, and with this algorithm, tuberculosis is diagnosed 63% of the time, which is less than the survey rate in all age groups in the same area, 81.2% of PTB cases [30]. The discrepancy could be due to the difference in population.The second algorithm is a cough < 2 weeks followed by a chest x-ray with findings suggestive of tuberculosis, followed by a GeneXpert/microscopic swab. Using this algorithm, we found 21% of tuberculosis cases, which is more than the study of all age groups in the same areas, accounting for 14.2% of PTB cases [30], this inequality is due to population defference.

The third algorithm was any-duration cough followed by GeneXpert/AFB swab microscopy, and with this algorithm we found 16%, more than one study in the same area across all ages, as 4.5% of PTB cases. This difference shows that GeneXpert provides more results in children with an indicative CXR score and is important in this age group. Chest X-ray was performed in 399/438 (91%), making it the most commonly used screening modality in this study. All chest radiographs were frontal (AP). Approximately 55/399 (13.78%) had evidence of tuberculosis on chest X-ray. Diagnosis was based on a cough screening algorithm followed by chest X-ray. Also, the algorithms required fewer modifications to be sentence specific. Thus, if a facility has an X-ray machine and a radiologist, this can be incorporated into the routine standard of care. As recommended by the World Health Organization, CXR screening is a good healthcare choice because it reduces costs and logistical challenges compared tocommunity-active case searches. Of 270/438 (61.6%),GeneXpert MTB/Ref gastric lavages were performed, of which 32 were positive, with a percentage positive of 11.8th %. Only 51 children had an AFB smear, of which 2 were positive with a positivity rate of 2/51 (3.9%).The use of cough screening algorithms followed by a chest x-ray followed by a GeneXpert sputum test resulted in 21% more cases of PTB than a chest x-ray alone (clinical) 13.78%. This result supports the recommendation to use GeneXpert as the first diagnostic test at the point-of-care [31, 32] over conventional tests due to its rapidity and sensitivity to diagnostic tests. resistant to TB. The use of cough screening was helpful in the COVID-19 time as TB services were compromised [33–35].

In this study, fever, weight loss, and TB contact history are highly associated with TB positivity rates in children. Differences in TB detection rates between countries such as Ethiopi a may be influenced by the prevalence of the HIV epidemic, overcrowding, differences in the sensitivity of laboratory diagnostic techniques, and variability in the effectiveness of preventive measures [36–40]. In current times where technologies are rapidly evolving to advance TB care and management [], such advanced technologies are are urgently needed to improve childhood TB case ditection. (Atehortúa et al., 2015; Hajizadeh et al., 2021).

## Conclusion

Microbiological pulmonary TB detection in children is low compared to clinical and x ray detection despite advancement in modern rapid test, such as GeneXpert making clinical the most sensitive diagnostic method in children. Fever ,weight loss and TB contact history is the most important clinical features that must be emphasized during clinical screening of TB in children. Socio-demographic history is important in our setting during clinical diagnosis of TB in children and must be given attention. Use the algorithm of combined clinical and confirmatory tests can improve rate of PTB diagnosis in children. We recommend use of symptom screening followed by CXR and the confirm by GeneXpert where the tests are available to improve microbiological diagnosis of childhood tuberculosis using more sensitive and specific diagnostic technique. In clinically high-risk children use of Chest x ray only is a good choice at health facilities that reduces the logistic challenges of bacteriological confirmation and possible under diagnosis. AFB smear is less important in children compared with GeneXpert to confirm TB case in children and not recommended especially for younger children less than 5 years.

## Figures and Tables

**Table 1:** Soci-demographic characteristics of participants and pattern of childhood TB diagnosis among presumptive TB cases in children at health facilities in Ethiopia

Variables	Pulmonary TB,n(%)	No TB,n(%)	P value

Age	10(23)	142(36)	<0.0001
1–5	20(46)	158(40)	
5–10	13(30)	95(24)	
10–14			

Gender	11(25.6)	194(49.2)	0.06
Male	32(74.4)	201(50.8)	
Female			

Address	29(67.4)	187(47.3)	<0.0001
Rural	14(32.6)	208(52.7)	
Urban			

**Table 2:** Clinical characteristics of study participants and pattern of TB diagnosis among suspected TB cases in children at public health facilities, Ethiopia

Variables	Childhood PTB,n(%)	No PTB,n(%)	P value

Fever	14(32.6)	193(48.9)	<0.0001
No	29(67.4)	202(51.1)	
Yes			

TB contact History	16(37.2)	181(45.8)	<0.0001
No	27(62.8)	214(54.2)	
Yes			

Blood in sputum	31(72.1)	391(99)	0.06
No	12(27.9)	4(1)	
Yes			

Weight loss	19(44.2)	224(56.7)	<0.0001
No	24(55.8)	171(43.3)	
Yes			

Night sweet	26(60.5)	326(82.6)	0.00346
No	3(7)	12(3)	
Not sure	14(32.5)	57(14.4)	
Yes			

Chronic disease	39(90.7)	393(99.5)	0.045
No	4(9.3)	2(0.5)	
Yes			

Cough duration	12(27.9)	176(44.6)	0.001
< 2weeks	31(72.1)	219(55.4)	
.>2 weeks			

AFB, Acid Fast Bacilli; TB, Tuberculosis; CXR,Chest X-ray

**Table 3:** Diagnostic algorithm and and pattern of TB diagnosis among suspected TB cases in children at public health facilities, Ethiopia

Algorithm	Childhood PTB, n(%)	No PTB, n (%)	P value

Cough > 2 weeks followed by CXR screening, then GeneXpert/AFB smear.	27(6.2)	204(56.6)	< 0.0001
Cough < 2 weeks followed by CXR screening, then GeneXpert/smear	9(2.1)	159(40.3)	
Cough of any duration followed by GeneXpert/ smear	7(1.5)	32(8.1)	

AFB, Acid Fast Bacilli; TB, Tuberculosis; CXR,Chest X-ray

**Table 4:** Factor that determine Pattern of Tuberculosis diagnosis in children at health facilities, Ethiopia

Variables	CPR	APR	Frequency(%)

Age	1	1	10(23)
1–5	4.6 (2.7–7.6)	2.2 (1.5–3.2)	20(46)
5–10	3.3 (1.9–5.8)	1.8 (1.2–2.6)	13(30)
10–14			

Gender	1	1	11(25.6)
Male	1.23 (1.0–1.5)	1.0 (0.9–1.1)	32(74.4)
Female			

Address	2.35(1.87–2.96)	1.3 (1.1–1.5)	29(67.4)
Rural	1	1	14(32.6)
Urban			

Night sweet	1.2 (1.1–1.3)	1.1 (0.9–1.2)	14(32.5)
Yes	1.4 (1.1–1.6)	1.2 (1.1–1.4)	26(60.5)
No	1	1	3(7)
Not sure			

Weight loss	2.3 (1.8–2.9)	1.1 (0.9–1.2)	24(55.8)
Yes	1	1	19(44.2)
No			

Blood in sputum	1	1	12(27.9)
Yes	1.5 (1.2–2.)	1.1 (0.9–1.3)	31(72.1)
No			

TB case contact history	1.6(1.1–1.6)	1.2 (0.9–1.4)	27(62.8)
Yes	1	1	16(37.2)
No			

Fever	1.8 (1.2–2.1)	1.3(1.0–1.4)	29(67.4)
Yes	1	1	14(32.6)
No			

CPR, Crude prevalence ratio; APR, Adjusted prevalence ratio;TB, Tuberculosis

## Data Availability

The datasets used and/or analyzed during the current study will be available from the corresponding author on reasonable request.
